# Comparison of GAL80ts and Tet-off GAL80 transgenes

**DOI:** 10.17912/micropub.biology.000770

**Published:** 2023-03-23

**Authors:** Taylor Barwell, Sofia Geld, Laurent Seroude

**Affiliations:** 1 Biology, Queen's University, Kingston, Ontario, Canada

## Abstract

The manipulation of gene expression requires complete control of space, time and level of expression. In *Drosophila* , the UAS/GAL4 system has been instrumental in achieving spatial control, but the pursuit for the ideal system for temporal control is ongoing. One strategy used by many groups is to use GAL80. Two different kinds of GAL80 transgenic constructs have been reported but have never been compared directly. Here, we characterize and compare the repression ability of the GAL80ts and Tet-off GAL80 transgenes by two different assays. We found that GAL80ts was generally more efficient than Tet-off GAL80 and that the level of repression was dependent on the GAL4 driver, and did not necessarily correlate with expression level. We also investigated the level of expression upon inactivation of GAL80ts. The level and kinetics of inactivation were found to differ depending on the GAL4 driver and the stage of the life cycle, and in many cases the inactivation was incomplete. These results emphasize that it is essential to measure experimentally the level of expression under repressed and unrepressed states when multiple GAL4 drivers are used with GAL80 transgenes.

**Figure 1. Comparative analysis of repression ability of GAL80ts and GAL80TET transgenes with Mef2-Gal4 (Mef2) and DJ694-Gal4 (DJ694):**
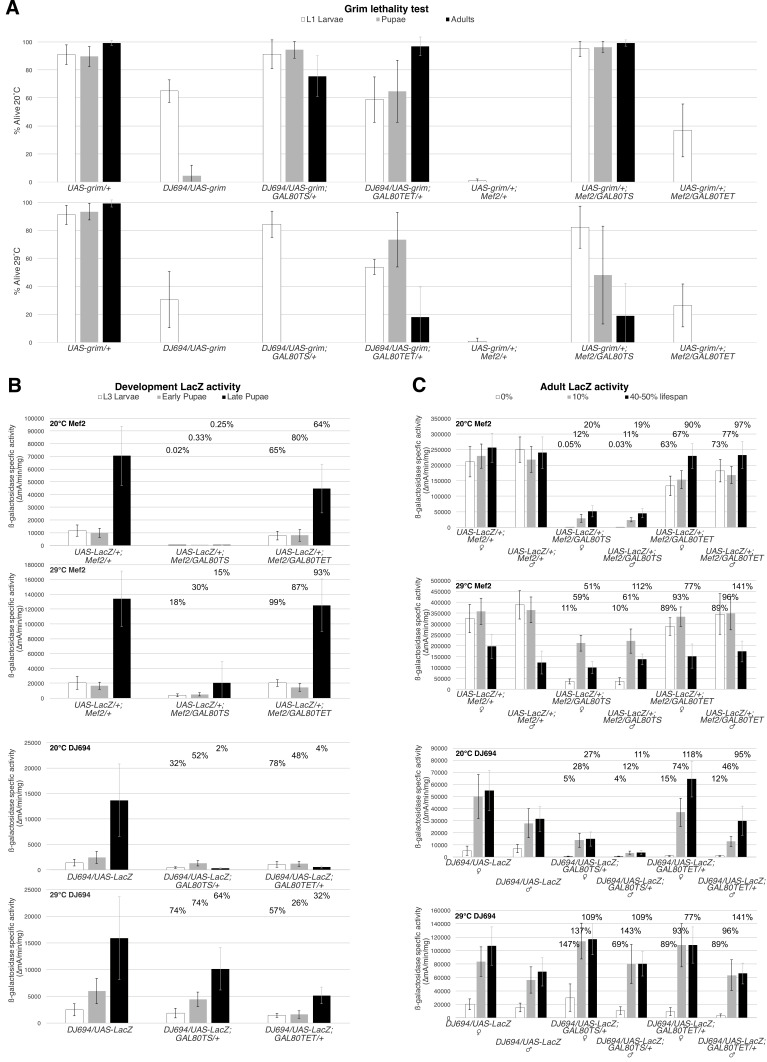
A) Grim developmental lethality assay. The y-axis shows the percentage of individuals alive relative to the previous stage. L1 larvae (white): percentage of eggs that hatched. Pupae (grey): percentage of L1 larvae that pupated. Adults (black): percentage of pupae that yielded adults. In the top graph animals were maintained at 20℃. In the bottom graph animals were maintained at 29℃. Error bars represent ± SD across all experimental replicates. B) Developmental expression of lacZ as measured by CPRG and Bradford assays. Y-axis shows the ß-galactosidase specific activity (∆mA/min/mg). Extracts were prepared from whole animal samples of third instar (L3) wandering larvae (white), early pupae (grey), late pupae (black). Bars represent the average of all four experimental replicates (5 extracts in each). Error bars represent ± SD across all experimental replicates. Animals were raised at either 20 or 29℃. The temperature and the driver used is indicated in the top left corner of each graph. The percentages atop each bar denote the percent expression relative to the no GAL80 control genotype. C) Adult expression of lacZ. Extracts were prepared from dissected thoraces. Bars represent the average of all four experimental replicates (5 extracts in each). Error bars represent ± SD across all experimental replicates. Different chronological time points were measured across the different temperature treatments in order to standardize to the physiological age. 0% lifespan (white): 20℃= 0-2d, 29℃= 0-2d. 10% lifespan (grey): 20℃= 10-12d, 29℃= 3-5d. 40-50% (black): 20℃= 40-42d, 29℃=15-17d. The percentages atop each bar denote the percent expression relative to the no GAL80 control genotype.

## Description

The ability to regulate gene expression in model organisms is essential to our understanding of fundamental biological processes. Complete control of gene expression requires the ability to manipulate spatial and temporal expression patterns, as well as the magnitude of expression. In *Drosophila* , spatial control of gene expression is achieved by the widely used UAS/GAL4 system (Brand and Perrimon, 1993) . Derived from yeast, the transcription factor GAL4 binds the upstream activation sequence (UAS) enhancer, activating transcription and driving expression of a gene of interest (Fischer et al., 1988) . This is an efficient, bipartite system in which the GAL4 and UAS transgenes are maintained as two separate fly lines and upon crossing, GAL4 will drive expression of the gene of interest in a pattern dictated by the GAL4 promoter. By increasing or decreasing the number of UAS sequences or by using alternate transcriptional activation domains (GAL4 variants, VP16, p65) it is possible to modulate the level of expression (Pfeiffer et al., 2010; Singh and Kim, 2022) . It is also possible to target any cell or tissue of interest by using two different transgenes to independently express the GAL4 DNA binding and transcriptional activation domains, leading to the activation of a UAS transgene only in the cells expressing both transgenes (Luan et al., 2006; Pfeiffer et al., 2010) . Many groups have engineered and introduced additional transgenes in order to add temporal control. One strategy used by several groups is to use GAL80 (Lai and Lee, 2006; Lee and Luo, 1999; McGuire et al., 2003; Suster et al., 2004) . GAL80 antagonizes GAL4 by inhibiting its activation domain, preventing interaction between GAL4 and the transcriptional machinery (Ma and Ptashne, 1987; Nogi and Fukasawa, 1989; Wu et al., 1996; Yun et al., 1991) . Thus, preventing expression of a UAS transgene. Here, we compare two different GAL80 constructs for their ability to repress GAL4-mediated gene expression. One transgene expresses GAL80ts, a thermosensitive GAL80 variant, under the control of a ubiquitous tubulin promoter (McGuire et al., 2003) . At the restrictive temperature 20℃, GAL80ts is active, and able to repress GAL4-mediated expression. At the permissive temperature 29℃, GAL80ts is unstable and repression is relieved. The other system is the Tet-off GAL80 transgenes (GAL80TET) (Barwell et al., 2017) . This is a two promoter construct in which GAL80 is under the control of a TetO promoter and a ubiquitous promoter drives expression of tTA (tetracycline transactivator molecule). As the name suggests, tTA is tetracycline sensitive. In the absence of tetracycline, tTA will bind TetO and activate expression of GAL80. In the presence of tetracycline, tTA will be inactive, thus there will be no GAL80 expression. Therefore, GAL4-mediated UAS expression can be temporally controlled by feeding tetracycline in the diet. We performed an extensive characterization of GAL80TET elsewhere (Barwell et al., 2017) . We found that optimal repression was achieved when two different versions of the transgene (each with a different ubiquitous promoter driving tTA), and two copies of each version were used. As well, we showed that the repression ability of GAL80 declined with age. While details of GAL80TET have been described, a direct comparison with the widely used GAL80ts transgene has not been done. In order to determine which transgene is ideal for control of gene expression, we measured repression ability by two different assays. Two previously described GAL4 drivers were used for these measurements, DJ694-Gal4 and Mef2-Gal4 (Martin et al., 2009; Ranganayakulu et al., 1998; Seroude et al., 2002) . These drivers were chosen because they both drive high expression and a very high ratio of GAL80 to GAL4 would overestimate repression. For GAL80TET, we used a copy of each version of the transgene inserted on the third chromosome. Since Mef2-Gal4 also resides on the third chromosome we only tested two copies.

In the first assay, the pro-apoptotic gene, *grim* , is driven by GAL4 which induces cell death in the GAL4 expressing cells (Chen et al., 1996) . If cell death is widespread enough or if the cells in which expression occurs are required for survival, the animal will die. This lethality is easily scored, and with the addition of the GAL80 transgenes the number of surviving individuals can be quantified as a measure of repression (Barwell et al., 2017; Poirier et al., 2008; Seroude, 2002) . In the second assay, a UAS-lacZ reporter is used to quantify ß-galactosidase specific activity. The use of a combination of two different assays serves to both validate and complement each other. For instance, if there is residual, unrepressed expression in cells that are not required for survival, it would go undetected by the grim assay but should be measured in the lacZ assay. Inversely, in some cell types the level of grim expression that causes lethality may be so low it is undetectable by the lacZ assay, but would be measurable with the grim assay.

Figure 1A shows the repression ability measured by the grim assay with two different GAL4 drivers, DJ694-Gal4 and Mef2-Gal4 at 20℃. With DJ694-Gal4, the addition of either GAL80 transgene results in an obvious increase in the number of surviving animals at all scored stages. However, GAL80ts leads to higher survival of L1 and pupae, whereas GAL80TET shows higher survival of adults. With Mef2-Gal4, GAL80ts results in complete absence of lethality at any stage, whereas GAL80TET only improved the survival of L1 larvae. With both drivers GAL80ts represses better than GAL80TET prior to the pupal stage but GAL80TET appears to repress better during the pupal stage with DJ694-Gal4. The assay was also performed at 29℃, which confirms the genotype as well as serves as an assessment of GAL80ts inactivation. Indeed at 29℃, the GAL80ts animals showed lower survival rates compared to the same genotype at 20℃. With DJ694-Gal4, the pupae and adults have been completely eliminated, however, the survival rate of L1 larvae was still relatively higher than the positive control that does not carry a GAL80 transgene. With Mef2-Gal4, the survival rate of all stages have been lowered but remain higher than the positive control, indicating that the inactivation is incomplete.

Figure 1B shows the repression ability measured by the lacZ assay during development. ß-galactosidase specific activity was measured in L3 larvae, early and late pupae. Overall, the GAL80ts transgene repressed better with both drivers than GAL80TET. This would suggest that the mutation conferring thermosensitivity may also confer greater repression ability. However, the repression ability was found to be highly dependent on the GAL4 driver that was used. While both drivers are expressed in the adult muscle, during development DJ694-Gal4 is primarily expressed in the oenocytes in the late pupae, while Mef2-Gal4 is expressed in the developing muscles from the third instar larval stage and through the pupal stages as well. The repression ability of GAL80TET appears to be more complete with DJ694-Gal4 (oenocytes) compared to Mef2-Gal4 (muscle). While with GAL80ts, it is the reverse, with better repression of Mef2-Gal4 than DJ694-Gal4. It is worth noting that the repression ability in the late pupae is similar between the two drivers with GAL80ts whereas GAL80TET shows a drastic difference between the two drivers. Although the difference with GAL80TET can be attributed to the Mef2-Gal4 having higher expression level than DJ694-Gal4, this cannot account for GAL80ts to be more efficient with Mef2-Gal4 than DJ694-Gal4. One reason why GAL80ts could work better with Mef2-Gal4 is the localization of GAL80. The expression of both GAL80 transgenes is dependent on the same ubiquitous *tubulin* promoter, but GAL80TET has another copy of the transgene that uses the *actin5c* promoter. In our previous work, it was shown that better repression was obtained by combining transgenes with two different promoters, rather than multiple copies of the same promoter. The combination of different versions of the transgene was found to cover a more widespread expression, particularly in the oenocytes (Barwell et al., 2017) . It is possible that GAL80ts is not highly expressed enough in the oenocytes to repress as well as in the muscle. It is also possible that GAL80ts shows reduced repression in oenocytes compared to muscle because of the tissue context where it is expressed. Measurements were also done at 29℃ to quantify levels of expression when GAL80ts is allegedly inactive. As predicted by the grim assay, ß-galactosidase activity increased but remained lower than the no GAL80 control, confirming that the inactivation of GAL80ts is incomplete with both drivers. The higher percentage of expression relative to the control with DJ694-Gal4 compared to Mef2-Gal4 accounts for the difference in survival observed in the grim assay. Although the complete elimination of pupae and adults with DJ694-Gal4 in the grim assay indicated complete inactivation, the lacZ assay reveals that this is not the case.

The repression ability was also measured in the adult thorax ( Figure 1C ). It is obvious that neither transgene completely blocks expression at all ages. With both drivers GAL80ts repressed better, resulting in lower residual expression than GAL80TET. The loss of repression with age is evident with both GAL80 molecules, although the decline is more pronounced with GAL80TET. This suggests that the GAL80ts mutation may also confer better stability at low temperature. It is evident that the level of repression is dependent on the GAL4 driver. GAL80TET decreased expression to a lower level with DJ694-Gal4 than Mef2-Gal4 in the thoracic muscles of the very young fly (0% of the lifespan). This difference is likely because DJ694-Gal4 has a lower expression level than Mef2-Gal4. It is also important to notice there are sex specific differences with DJ694-Gal4 but not Mef2-Gal4. Again we observe that the GAL80ts transgene repressed Mef2-Gal4 better than DJ694-Gal4 in youngest animals despite the driver being higher expressed. This difference cannot be attributed to a difference between oenocytes and muscles since extracts were prepared from dissected thoraces. The measurements at 29℃ revealed drastic driver specific differences in the level and kinetics of inactivation with GAL80ts. The kinetics with Mef2-Gal4 is much slower than with DJ694-Gal4. Once they reach adulthood, the ß-galactosidase activity is comparable to the control without GAL80 with DJ694-Gal4, while it is ten times lower than the control with Mef2-Gal4. In addition, at no age does the Mef2-Gal4 activity reach the level of the control, except for the oldest males.

We conclude by pointing out that the experimental data of the controls (no GAL80) can also be used to examine the effect of temperature on GAL4 activity. It is known that GAL4 activity increases with temperature but it is also dependent on the driver used (Seroude et al., 2002) . Indeed our data shows that GAL4 activity increases with temperature and there are obvious differences depending on the driver used or the stage of the life cycle. With both drivers, the increase in temperature results in approximately a doubling of expression level in the L3 larvae and early pupae. In contrast, in the late pupae a similar increase is seen with Mef2-Gal4 while a marginal change is observed with DJ694-Gal4. In the adult, the expression level approximately doubles at all ages with DJ694-Gal4, whereas with Mef2-Gal4, an approximately 50% increase is observed in the young animals and a decrease is seen in the oldest animals.

## Methods

Drosophila strains and culture

Flies were maintained on standard cornmeal medium (0.01% molasses, 8.2% cornmeal, 3.4% yeast, 0.94% agar, 0.18% benzoic acid, 0.66% propionic acid), which was prepared as described (Barwell et al., 2022) , at either 20℃ or 29℃ as indicated.

The following lines were used for this study: *w ^1118^* , DJ694-Gal4 *w ^1118^ ; P{w ^+mW.hs^ =GawB}EDTP ^DJ694^* (BDSC: 8176) (Seroude et al., 2002) , Mef2-Gal4 (BDSC: 27390) (Ranganayakulu et al., 1998) , UAS-grim *w ^1118^ ; P{w ^+mC^ =UAS-grim.N-2}* (obtained from J Abrams) (Wing et al., 1998) , UAS-lacZ *w ^+^ ; P{w ^+mC^ =UAS-lacZ.B} ^Bg4-1-2^* (BDSC:1776), GAL80ts *w ^+^ ; P{w ^+mC^ =tubP-GAL80 ^ts^ }2/TM2* (BDSC:7017), Tet-off GAL80 transgenes, in short, GAL80TET (Barwell et al., 2017) .

The following recombinant lines were generated here by standard genetic crosses:

-UAS-grim; GAL80ts

-UAS-grim; GAL80 ^3.3,DJ1077^

-UAS-lacZ;GAL80ts

-UAS-lacZ;GAL80 ^3.3,DJ1077^

UAS-grim lethality assay

The complete dataset and experimental workflow diagram are available in Extended Data and have been deposited to Zenodo ( https://doi.org/10.5281/zenodo.7555938 ). Experimental genotypes were obtained by crossing UAS-grim, UAS-grim;GAL80ts, UAS-grim;GAL80 ^3.3,DJ1077^ with DJ694-Gal4 and Mef2-Gal4. A negative control was obtained by crossing UAS-grim with w ^1118^ . Parents were obtained from multiple independent cultures to set up independent crosses. Biological replicate crosses were obtained from independent sets of parents. Crosses were maintained and eggs were collected at 20℃. Crosses were kept in culture tubes for 24-48h to allow for mating before transferring them to egg collectors. Multiple egg collections were done from each parental set (Technical replicates).

The egg collectors were fashioned from empty vials (with air holes) and small plastic medicine cups, that could fit onto and seal the vials. Egg collector medium was distributed to the cups (2% agar, 5% sucrose, dyed with neutral red). Once medium was set, a small dollop of yeast paste (yeast with water until peanut butter consistency) was piped onto the surface of the medium using a syringe. At least 25 females and 25 males were used for each cross and allowed to mate overnight for 12-16h. Breaking open the side of a vial of standard fly food, circular disks were sliced (~0.5cm thick), from the cylinder of food and transferred onto a flat, clean surface to align eggs. Up to 25 embryos were aligned per slice, and then the slice with eggs was transferred on top of a standard vial of food to continue development at either 20℃ or 29℃. Up to 4 slices were used for a given collection from a set. The scoring of the number of first-instar larvae (L1) was done by scoring the number of empty eggs 25-30h (29˚C) or 43-48h (20˚C) after egg alignment. The number of pupae and adults was scored 5-6 days (29˚C) or 8-10 days (20˚C) after the scoring of the previous stage.

UAS-lacZ CPRG assay

The complete dataset and experimental workflow diagram are available in Extended Data and have been deposited to Zenodo ( https://doi.org/10.5281/zenodo.7555300 ). Experimental genotypes were obtained by crossing UAS-lacZ, UAS-lacZ;GAL80ts, UAS-lacZ;GAL80 ^3.3,DJ1077^ with DJ694-Gal4 and Mef2-Gal4. Crosses were set up in duplicate, one raised at 20℃ and the other at 29℃. Measurements of ß-galactosidase specific activity were performed as described (Barwell and Seroude, 2022) . Extracts were prepared from whole third instar (L3) larvae, early (yellow) pupae, and late (dark) pupae, sex was not determined. Extracts were also prepared from male and female adult dissected thoraces. Since different temperatures were being compared, flies were collected and staged to equate physiological age at early, midlife and late life time points. At 20℃, 0% of lifespan is equivalent to a chronological age of 0-2d, 10% is 10-12d, 40% is 40-42d. At 29℃, 0% is equivalent to a chronological age of 0-2d, 10% is 3-5d, 50% is 15-17d. Four experimental replicates were measured per genotype with 5 individuals measured in each. Replicate 1 and 2 were started simultaneously by setting up separate replicate cultures. Replicate 3 and 4 were set up 2 days later by transferring the parents from the Replicate 1 and 2 cultures respectively into new vials.

## Reagents

**Table:** 

**REAGENT**	**DESCRIPTION**	**SOURCE**
DJ694-Gal4	* w ^1118^ ; P{w ^+mW.hs^ =GawB}EDTP ^DJ694^*	BDSC: 8176
Mef2-Gal4	* y ^1^ w ^+^ ; P{w ^+mC^ =GAL4-Mef2.R} ^3^*	BDSC: 27390
UAS-grim	* w ^1118^ ; P{w ^+mC^ =UAS-grim.N} ^2^*	(Wing et al., 1998)
UAS-lacZ	* w ^+^ ; P{w ^+mC^ =UAS-lacZ.B} ^Bg4-1-2^*	BDSC:1776
GAL80ts	* w ^+^ ; P{w ^+mC^ =tubP-GAL80 ^ts^ } ^2^ /TM2 *	BDSC:7017
GAL80 ^3.3,DJ1077^	* w ^1118^ ; P{w ^+mC^ =TetO-GAL80 tubP-tTA} ^DJ1077^ ,P{w ^+mC^ =TetO-GAL80 act5CP-tTA} ^3.3^*	(Barwell et al., 2017)

## Data Availability

Description: CPRG workflow and dataset. Resource Type: Dataset. DOI: https://doi.org/10.22002/7vge7-ryk74 Description: Grim workflow and dataset. Resource Type: Dataset. DOI: https://doi.org/10.22002/jcfp4-zjm73
